# HSP47 in Mitochondria: Roles in Apoptosis, Signal Transduction, and Protein and Virus Transportation

**DOI:** 10.3390/cells15171601

**Published:** 2026-09-02

**Authors:** Sirirat Surinkaew, Noriko Hosoya, Hiromu Ito, Hiroshi Abe, Ikuo Nakanishi, Ken-ichiro Matsumoto, Motoki Imai, Fumitaka Kawakami, Makoto Kubo, Takafumi Ichikawa, Hiroshi Ichikawa, Yoshikazu Yonei, Hisashi J. Beppu, Junko Suka, Rachasak Boonhok, Moragot Chatatikun, Suriyan Sukati, Nitita Piya-amornphan, Chadapa Rungruangbaiyok, Charupa Lektip, Voravuth Somsak, Luksana Chaiswing, Pradoldej Sompol, Jun Urano, Meng Ling Moi, Hiroshi Takayanagi, Hiroko P. Indo, Hideyuki J. Majima

**Affiliations:** 1 School of Allied Health Sciences, Walailak University, Nakhon Si Thammarat 80160, Thailand; sirirat.sr@wu.ac.th (S.S.); rachasak.bo@mail.wu.ac.th (R.B.); suriyan.su@wu.ac.th (S.S.); charupa.le@wu.ac.th (C.L.); voravuth.so@wu.ac.th (V.S.); 2 Research Excellence Center for Innovation and Health Products (RECIHP), School of Allied Health Sciences, Walailak University, Nakhon Si Thammarat 80160, Thailand; 3 Laboratory of Molecular Radiology, Center for Disease Biology and Integrative Medicine, Graduate School of Medicine, The University of Tokyo, Tokyo 113-8655, Japan; nhosoya@m.u-tokyo.ac.jp; 4 Department of Oncology, Graduate School of Medical and Dental Sciences, Kagoshima University, Kagoshima 890-8544, Japan; 5 Quantum RedOx Chemistry Team, Quantum Life Spin Group, Institute for Quantum Life Science (iQLS), National Institutes for Quantum Science and Technology (QST), 4-9-1 Anagawa, Chiba 263-8555, Japan; nakanishi.ikuo@qst.go.jp; 6 Quantitative RedOx Sensing Group, Department of Radiation Regulatory Science Research, Institute for Radiological Science (NIRS), National Institutes for Quantum Science and Technology (QST), 4-9-1 Anagawa, Chiba 263-8555, Japan; matsumoto.kenichiro@qst.go.jp; 7 Department of Molecular Diagnostics, School of Allied Health Sciences, Kitasato University, 1-15-1 Kitasato, Sagamihara 252-0373, Japan; 8 Department of Applied Tumor Pathology, Graduate School of Medical Sciences, Kitasato University, Sagamihara 252-0374, Japan; 9 Regenerative Medicine and Cell Design Research Facility, School of Allied Health Sciences, Kitasato University, 1-15-1 Kitasato, Sagamihara 252-0373, Japan; kawakami@kitasato-u.ac.jp (F.K.);; 10 Department of Regulation Biochemistry, Graduate School of Medical Sciences, Kitasato University, 1-15-1 Kitasato, Sagamihara 252-0373, Japan; 11 Department of Health Administration, School of Allied Health Sciences, Kitasato University, 1-15-1 Kitasato, Sagamihara 252-0373, Japan; 12 Department of Environmental Microbiology, Graduate School of Medical Sciences, Kitasato University, 1-15-1 Kitasato, Minami-ku, Sagamihara 252-0373, Japan; 13 Department of Medical Life Systems, Graduate School of Life and Medical Sciences, Doshishia University, Kyoto 610-0394, Japan; 14 Anti-Aging Medical Research Center and Glycation Stress Research Center, Graduate School of Life and Medical Sciences, Doshisha University, Kyoto 610-0394, Japan; 15 Dr. Beppu’s Oral Health Care & Anti-Aging Clinic, Iwakami Building 3F, 3-2-6 Nihonbashi, Chuo-ku, Tokyo 103-0027, Japan; 16 Department of Medical Technology and Science, Faculty of Health Science, Kyoto Tachibana University, 34 Yamada-cho Otake, Yamashina-ku, Kyoto 607-8175, Japan; 17 Hematology and Transfusion Science Research Center (HTSRC), School of Allied Health Sciences, Walailak University, Nakhon Si Thammarat 80160, Thailand; 18 Department of Toxicology and Cancer Biology, 454 Bosomworth HSRB, University of Kentucky College of Medicine, Lexington, KY 40536, USA; 19 Sanders-Brown Center on Aging, University of Kentucky College of Medicine, Lexington, KY 40536, USA; 20 Cellibre, San Diego, CA 92121, USA; 21 School of International Health, Graduate School of Medicine, The University of Tokyo, Tokyo 113-0033, Japan; 22 Department of Immunology, Graduate School of Medicine and Faculty of Medicine, The University of Tokyo, Tokyo 113-8655, Japan; 23 Department of Microbiology and Immunology, Institute for Immunological Sciences, Department of Orthopaedics, University of Rochester Medical Center, 601 Elmwood Avenue, Rochester, NY 14642, USA; 24 Amanogawa Galactic Astronomy Research Center (AGARC), Graduate School of Sciences and Engineering, Kagoshima University, 1-21-40 Korimoto, Kagoshima 890-0065, Japan

**Keywords:** mitochondria, mitochondrial reactive oxygen species (mtROS), HSP47, oxidative stress, apoptosis, signal transduction

## Abstract

**Highlights:**

- **HSP47 translocates from the endoplasmic reticulum to mitochondria, serving as an essential factor for both cellular and viral protein transportation.**
- **This study reveals that HSP47-bound, mitochondria-targeted proteins induce DNA damage, ROS generation, and specific signaling pathways, highlighting their potential in modulating cellular responses.**

**Abstract:**

Mitochondria are essential organelles for cellular energy production and the regulation of diverse biological processes, including apoptosis, redox homeostasis, and intracellular signaling. Although mitochondrial reactive oxygen species (mtROS) act as critical mediators of these functions, the molecular mechanisms underlying mtROS regulation remain poorly understood. This review summarizes current insights into the role of heat shock protein 47 (HSP47) in mitochondrial oxidative stress and mtROS-mediated cellular responses. In addition to its classical function as an endoplasmic reticulum (ER) chaperone, HSP47 translocates to the mitochondria under oxidative stress conditions. This mitochondrial localization promotes mtROS production, thereby triggering apoptotic pathways and redox-sensitive signal transduction. Furthermore, we examine the mechanistic insights linking HSP47 to mitochondrial function and oxidative stress, highlighting their implications for cellular homeostasis and disease pathogenesis. Overall, these findings establish HSP47 as a novel regulator of mtROS generation, suggesting that the HSP47–mtROS axis represents a promising therapeutic target for oxidative stress-related disorders and a potential role for exploring virus-induced cellular responses in future research.

## 1. Origin of Mitochondria

Earth formed ~4.6 billion years ago, establishing conditions for life’s emergence and evolution [1,2]. Early life evolved from prokaryotic ancestors, with endosymbiosis playing a crucial role in eukaryotic development [2,3]. Notably, mitochondria originated ~1.5–1.8 billion years ago when an ancestral bacterial cell entered a stable symbiotic relationship within an archaeal host cell [4]. A central debate in eukaryogenesis concerns the precise timing of this mitochondrial acquisition. Recently, Martijn et al. (2018) challenged the traditional view of a Rickettsiales or alphaproteobacterial origin, proposing instead that mitochondria evolved from an ancestral proteobacterial lineage that diverged prior to all known alphaproteobacteria [5]. This endsymbiotic event coincided with rising atmospheric oxygen levels, which profoundly shaped evolution. Although oxygen enabled highly efficient aerobic energy production via oxidative phosphorylation—generating far more ATP than anaerobic glycolysis—its reactive nature also produced damaging reactive oxygen species (ROS) [6,7,8,9,10]. Consequently, cells evolved diverse enzymatic and non-enzymatic antioxidant defense systems to maintain redox homeostasis [6,7,8,9,10]. Ultimately, this enhanced bioenergetic capacity facilitated the evolution of larger, complex multicellular organisms, leading much later to modern humans in Africa ~120,000 years ago [11]. Thus, humans would not exist today if mitochondria had not successfully integrated into cells.

## 2. Mitochondrial Protein Import and Intracellular Trafficking

### 2.1. Mitochondrial Genome and Nuclear-Encoded Proteins

Mitochondria are essential for numerous cellular processes, including oxidative phosphorylation, metabolic pathways, and programmed cell death [12]. To perform these diverse functions, they rely on the import of approximately 1000 cytosolically synthesized precursor proteins, making efficient protein import critical for mitochondrial biogenesis and maintenance. Depending on the species, mitochondria contain between 600 and over 2000 distinct proteins distributed across four structural subcompartments [13]. In humans, despite this complexity, only 13 of the roughly 600 mitochondrial proteins are encoded by mitochondrial DNA (mtDNA) [14]. This 16,569-base-pair circular molecule also encodes 22 tRNAs and 2 rRNAs. Unlike nuclear-encoded proteins, these 13 proteins are synthesized directly within the organelle, where mtDNA exists in multiple copies and can reach thousands of copies per cell [14].

### 2.2. Mitochondrial Protein Import Machinery

Most mitochondrial proteins are nuclear-encoded and imported into mitochondria post- or co-translationally [15]. Mitochondrial biogenesis relies on sorting roughly 1000 precursor proteins via sophisticated translocation systems that recognize targeting signals [16]. The translocase of the outer membrane (TOM) complex serves as the primary entry gate, where Tom20/Tom22 receptors and the Tom40 channel translocate proteins with N-terminal sequences [15,16]. Proteins then diverge into distinct pathways. The TIM23 complex, driven by membrane potential (Δψ) and the ATP-dependent PAM motor, transports N-terminal presequence proteins [15]. Alternatively, the TIM22 complex inserts multi-pass membrane proteins with internal signals into the inner membrane [16]. Together with SAM and MIA pathways, these complexes form an integrated network essential for mitochondrial biogenesis and cellular homeostasis [17].

For most mitochondrial proteins, targeting is mediated by a cleavable, N-terminal mitochondrial targeting sequence (MTS) that forms a positively charged amphipathic α-helix. This sequence is recognized by the TOM and TIM complexes during import [18,19] and is subsequently removed by mitochondrial processing peptidase (MPP) to generate the mature protein [20,21,22]. The identification of these proteins has been enhanced by bioinformatic tools like MTSviewer [23]. Key examples of MTS-containing proteins include the antioxidant enzymes MnSOD (SOD2) [10] and GPx4 [24,25,26], both of which are crucial for maintaining mitochondrial redox homeostasis. In contrast, proteins destined for the endoplasmic reticulum (ER) utilize N-terminal ER signal peptides—comprising a charged region, a hydrophobic core, and a cleavage site—which are recognized by the signal recognition particle (SRP) for ER translocation [27,28,29]. Although both function as intracellular targeting signals, MTSs and ER signal peptides differ significantly in their sequence features, structural characteristics, and recognition mechanisms.

### 2.3. Mitochondrial Targeting Sequences (MTSs) and Organelle-Specific Protein Trafficking

Accurate delivery of nuclear-encoded proteins into mitochondria requires a targeting signal within the precursor protein and a cellular transport machinery that recognizes this signal to direct the protein to its destination [18,19].

For most mitochondrial proteins, targeting is mediated by a cleavable N-terminal mitochondrial targeting sequence (MTS) that forms a positively charged amphipathic α-helix. This sequence is recognized by TOM complex receptors and transferred to the TIM machinery for import [18,19], after which it is typically removed by mitochondrial processing peptidase (MPP) to generate the mature protein [20,21,22]. The identification of these numerous MTS-containing proteins has been enhanced by bioinformatic resources like the MTSviewer database [23]. Key examples include manganese superoxide dismutase (MnSOD, SOD2), an antioxidant enzyme protecting against oxidative damage, and phospholipid hydroperoxide glutathione peroxidase (GPx4), which utilizes its N-terminal sequence for mitochondrial localization and redox homeostasis maintenance [24,25,26].

Proteins destined for the ER contain N-terminal signal peptides, featuring a charged region, a hydrophobic core, and a cleavage site, that direct them to the secretory pathway [27]. Unlike mitochondrial targeting signals (MTSs), these ER signal peptides are recognized by a signal recognition particle (SRP) and directed to the ER translocation machinery [28,29].

## 3. Mitochondrial ROS Generation During Energy Production

### 3.1. The Electron Transport Chain (ETC)

Mitochondria are the primary energy-producing organelles in eukaryotic cells and play a central role in maintaining cellular homeostasis. In the presence of oxygen, mitochondria can generate 38 ATP from glucose [9,10]. This is in comparison to only two ATPs that can be produced solely through glycolysis in the absence of oxygen [30]. In the atmosphere, a reaction of hydrogen and oxygen into water vapor releases 242 kJ/mol of heat [31]. In cells, the mitochondrial ETC produces 473 kJ/mol from glucose and O_2_ [10]. This 473 kJ/mol is almost two times more energy production compared to heat generation (242 kJ/mol) from a reaction of hydrogen and oxygen into water vapor [31]. Glycolysis produces 96 kJ/mol from one glucose [30]. The additional energy of 473 kJ/mol from glucose is generated by a process known as oxidative phosphorylation, where electrons generated through the citric acid cycle from glucose breakdown products are funneled through the ETC and transferred to oxygen to produce water and ATP [32].

### 3.2. Generation of Superoxide Through the Electron Transport Chain

Aerobic energy production is accompanied by the inevitable generation of ROS. ROS comprise a group of oxygen-derived reactive molecules, including superoxide anion (O_2_^•−^), hydrogen peroxide (H_2_O_2_), and hydroxyl radicals (^•^OH). In addition, reactive nitrogen species (RNS) contribute to cellular redox regulation and oxidative stress. Among these reactive species, hydroxyl radicals are highly reactive and capable of causing extensive biomolecular damage, whereas superoxide anions exhibit lower reactivity but serve as important precursors for the generation of other ROS [9,10].

Mitochondrial oxidative phosphorylation is the principal intracellular source of ROS under physiological conditions. Oxidative phosphorylation is highly efficient in energy production, generating substantially more ATP than anaerobic glycolysis [30]. However, the ETC is not completely efficient, and a small portion of electrons can prematurely escape from the electron transport pathway. These leaked electrons react directly with molecular oxygen, resulting in the formation of O_2_^•−^, which represents the primary ROS generated within mitochondria [10]. Earlier studies estimated that approximately 2–3% of electrons undergo such leakage during mitochondrial respiration, although the extent of electron leakage varies depending on cell type, metabolic state, and physiological conditions [10,33,34,35,36].

**Figure 1:**
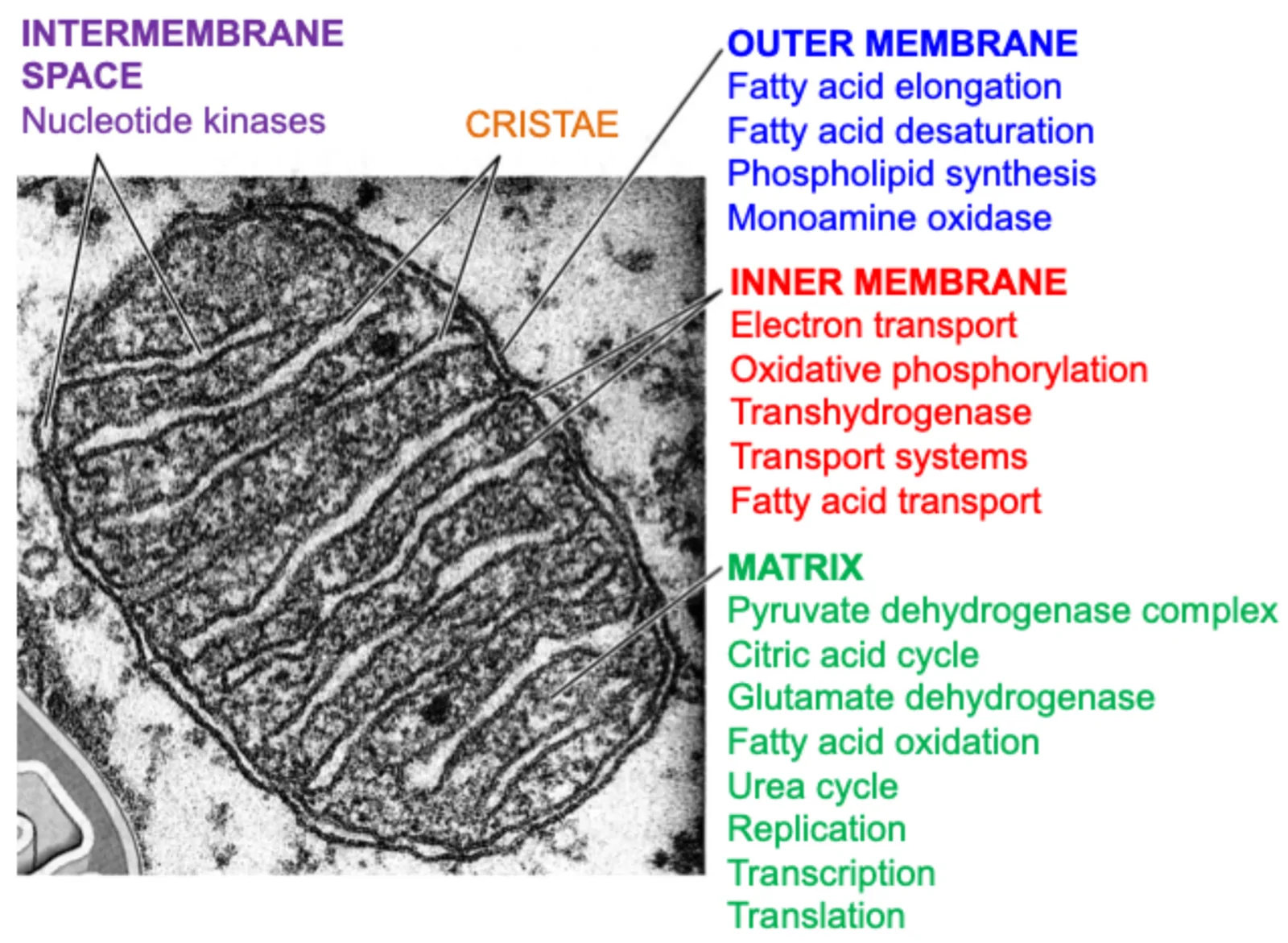
**Structure and major biological functions of mitochondria.** Representative electron micrograph showing the outer mitochondrial membrane, intermembrane space, and cristae. The electron transport chain (ETC), located within the inner mitochondrial membrane, generates ATP through oxidative phosphorylation and produces mtROS through electron leakage. In addition to energy production, mitochondria regulate apoptosis, intracellular signal transduction, calcium homeostasis, thermogenesis, and cellular metabolism. Adapted from Mathews CK and van Holde KE, Biochemistry, 4th Edition [37].

**Figure 2:**
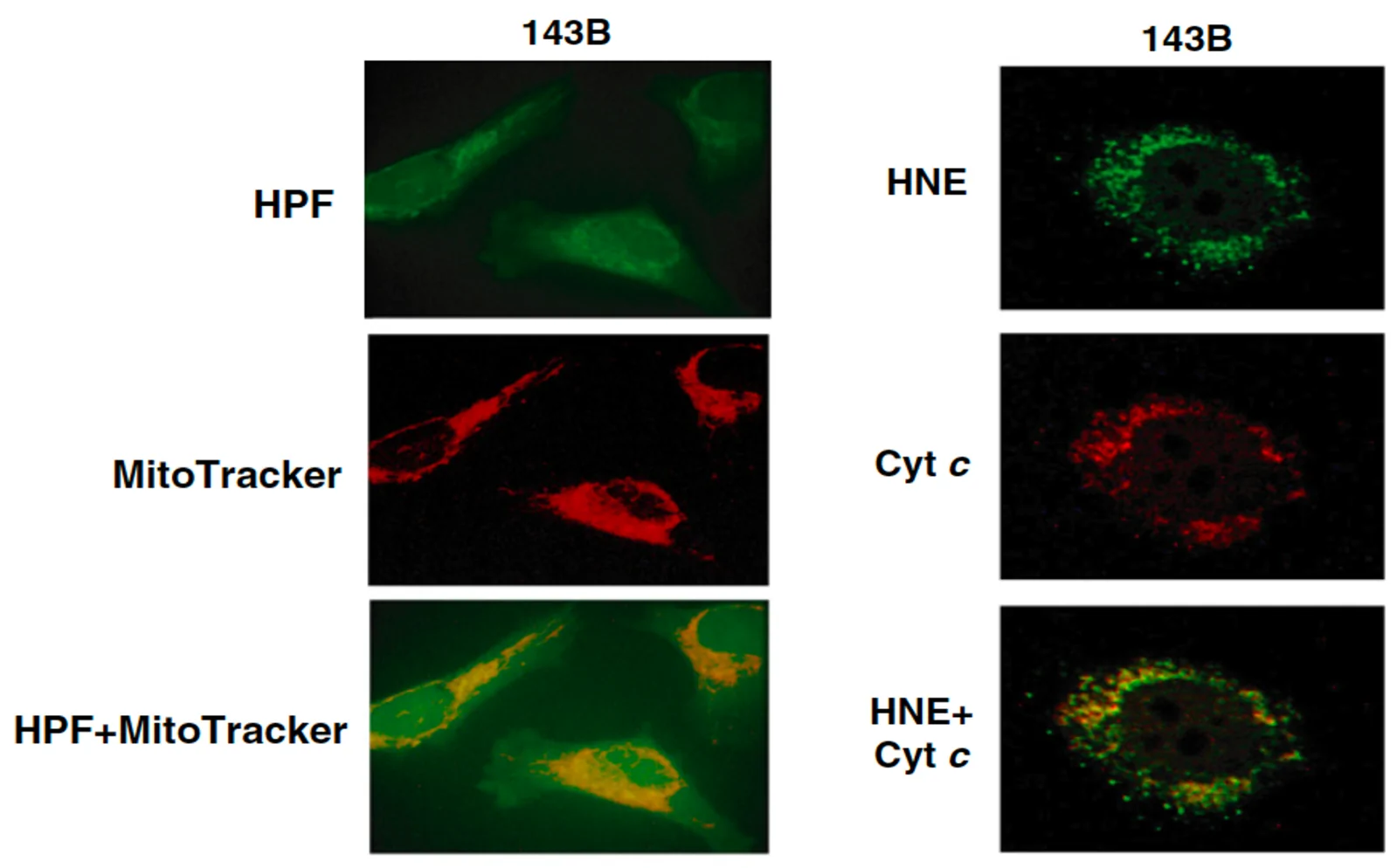
**Most intracellular ROS are generated by mitochondria, and lipid peroxidation occurs around mitochondria**. (**left**) Representative images of live 143B intact cells in which ROS were visualized with HPF at 37 °C. To examine the location of mitochondria, the same cells were stained with MitoTracker Red CMXRos and visualized. Merged double images of HPF and MitoTracker fluorescence were constructed to identify ROS in mitochondria. Most ROS were localized in mitochondria, as shown by the yellow color (green plus red) in the merged images for the cells. (**right**) 143B intact cells were fixed, and a major lipid oxidation product, HNE, was immunocytochemically stained. To localize HNE (green color), double staining was performed using anti-cytochrome *c* (Cyt c), which is localized in mitochondria (red color). Most HNE was localized in mitochondria, as seen in the merged image (yellow color). Modified from Indo et al. 2007 [38].

Figure 1 shows the structure and major biological functions of mitochondria. Figure 2 shows the mitochondrial localization of reactive oxygen species and lipid peroxidation following irradiation-induced stress. Superoxide production occurs predominantly at complexes I and III. Under physiological conditions, mitochondrial antioxidant systems rapidly detoxify superoxide and maintain redox homeostasis. Among these defense mechanisms, manganese superoxide dismutase (MnSOD) plays a central role by catalyzing the conversion of superoxide into hydrogen peroxide, which can subsequently be metabolized by catalase, glutathione peroxidases, and other antioxidant enzymes [10].

When ROS generation exceeds the capacity of antioxidant defenses, mitochondrial ROS (mtROS) accumulate and induce oxidative stress. Elevated mtROS levels can damage mitochondrial lipids, proteins, and DNA, leading to lipid peroxidation and amplification of oxidative chain reactions within the cell [10].

### 3.3. The Mitochondrial Superoxide Theory

The biological significance of superoxide became evident following the discovery of superoxide dismutase (SOD) by McCord and Fridovich in 1969 [39]. Subsequently, Weisiger and Fridovich identified MnSOD, a mitochondrial isoform that catalyzes the conversion of superoxide to hydrogen peroxide within the mitochondrial matrix [40]. These findings established the importance of superoxide metabolism in cellular physiology and defense against oxidative stress.

Based on these observations, Fridovich proposed the superoxide theory of oxygen toxicity, which postulated that superoxide anions are key mediators of oxygen toxicity and contribute to aging and a variety of degenerative diseases [41]. Building upon the superoxide theory of oxygen toxicity proposed by Fridovich, subsequent studies have highlighted mitochondria as a major intracellular source of superoxide. These observations led to the development of the mitochondrial superoxide theory [10], which proposes that mitochondrial superoxide is not merely a byproduct of aerobic metabolism but also a critical mediator of cellular signaling, apoptosis, aging, and disease processes [10].

## 4. Mitochondrial ROS as Mediators of Apoptosis

The recognition of mitochondria as central regulators of apoptosis emerged during the mid-1990s. Prior to this period, mitochondria were primarily viewed as cellular powerhouses responsible for ATP production. However, accumulating evidence revealed that mitochondria also play critical roles in the regulation of programmed cell death. Zamzami et al. (1995) demonstrated a close association between alterations in mitochondrial membrane potential and the induction of apoptosis, providing one of the earliest indications that mitochondria actively participate in cell death pathways [42].

A major breakthrough occurred in 1996 when cytochrome *c*, a component of the mitochondrial ETC, was identified as a key mediator of apoptosis following its release from mitochondria into the cytosol [43]. Subsequent studies further established the importance of mitochondrial outer membrane permeabilization in apoptotic signaling. In particular, B-cell/CLL lymphoma 2 (Bcl-2) family proteins were shown to regulate the release of cytochrome c by controlling the permeability of the mitochondrial outer membrane, thereby determining cellular susceptibility to apoptosis [44].

In parallel with these discoveries, increasing attention was directed toward the role of mtROS in apoptotic processes. Majima et al. (1998) demonstrated that cells overexpressing MnSOD exhibited resistance to apoptosis induced by mtROS [6,7,8]. These findings provided direct evidence that superoxide generated within mitochondria contributes to apoptotic signaling and supported the concept that mtROS function as active mediators of cell death rather than passive byproducts of oxidative metabolism. These observations further strengthened the mitochondrial superoxide theory, which emphasizes the central role of mitochondrial superoxide in regulating cellular fate [9].

Additional support for the involvement of mtROS in apoptosis was provided by Arai et al. (1999), who reported that mitochondrial phospholipid hydroperoxide glutathione peroxidase (GPx4) protects cells against oxidative injury and suppresses mitochondrial oxidative damage [45]. Together, these findings highlighted the importance of mitochondrial antioxidant systems in determining cellular responses to oxidative stress and apoptosis.

**Figure 3:**
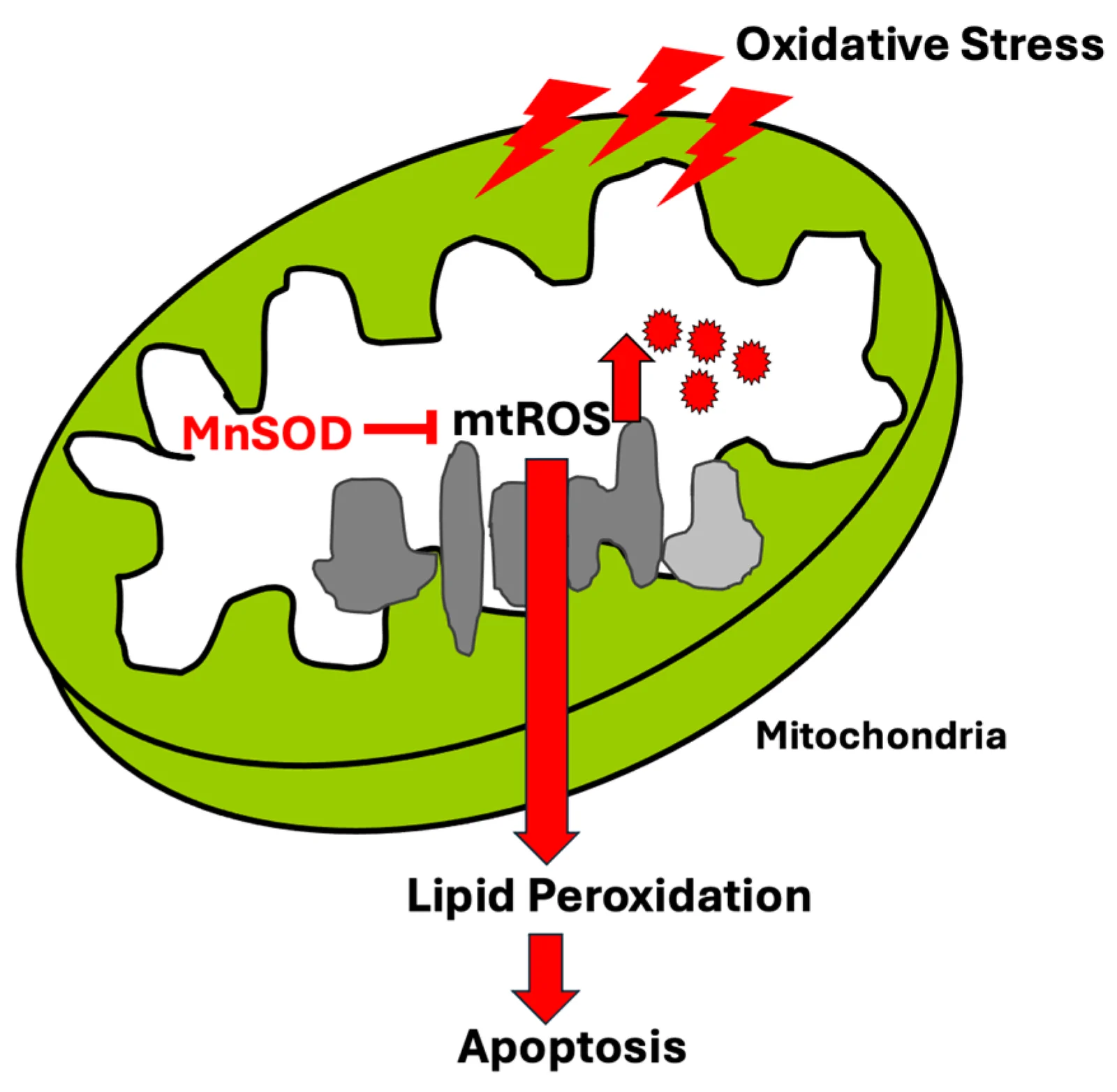
**The role of mtROS in oxidative stress-induced apoptosis.** Oxidative stress increases mtROS production, primarily through electron leakage from the ETC. Elevated mtROS promote lipid peroxidation and mitochondrial dysfunction, ultimately leading to apoptotic cell death. MnSOD suppresses mtROS accumulation by catalyzing the conversion of superoxide to hydrogen peroxide, thereby limiting oxidative damage and apoptosis. Adapted from Majima et al. 1998 [6].

Mitochondria-mediated apoptosis is now recognized as a highly regulated process involving multiple mitochondrial factors. In addition to cytochrome c, mitochondria contain several pro-apoptotic molecules, including apoptosis-inducing factor (AIF) [46] and proteins involved in caspase activation pathways [47,48]. During oxidative stress, increased mtROS production can promote mitochondrial membrane dysfunction, alterations in calcium homeostasis, and the release of these apoptotic factors into the cytosol. Consequently, mtROS serve not only as initiators of oxidative damage but also as critical signaling molecules that connect mitochondrial dysfunction to the activation of programmed cell death pathways (Figure 3).

## 5. Mitochondrial ROS (mtROS) as Mediators of Signal Transduction

### 5.1. Can mtROS Exit the Organelle?

mtROS originate primarily from O_2_^•−,^ which is generated following electron leakage from the ETC. Superoxide subsequently gives rise to a variety of ROS and RNS, including ^•^OH, singlet oxygen (^1^O_2_), H_2_O_2_, hydroperoxyl radicals (HO_2_^•^), nitric oxide (NO^•^), nitrogen dioxide (NO_2_^•^), peroxynitrite (ONOO^−^), and peroxynitrous acid (ONOOH) [10]. A critical question in mitochondrial redox biology is whether ROS generated within mitochondria can cross mitochondrial membranes and directly participate in cytosolic signal transduction.

To address this issue, we previously evaluated the membrane permeability of various ROS and RNS using calculated dipole moments and experimentally determined permeability coefficients [49]. Membrane permeability is influenced by several physicochemical properties, including molecular size, electrical charge, and dipole moment [49,50,51]. The dipole potential (represented by Ψd) is defined as the potential difference that arises due to the nonrandom orientation of dipolar residues of the lipids and associated water molecules within the membrane [52,53]. Molecules with electrical charges have difficulty passing through mitochondrial membranes due to their large number of dipoles [54]. The results of the dipole moments show that H_2_O_2_ is permeable (the dipole moment is 0.00 D). The dipole moment of NO_2_^•^ was 0.35 D, indicating permeability. Although the dipole moment of O2^•−^ is 0.00 D, the negative charge in O_2_^•−^ precludes its penetration into the membrane. ONOO^–^ is non-permeable. H_2_O (with a dipole moment of 1.89 D), ^•^OH (with a dipole moment of 1.67 D), ONOOH (with a dipole moment of 1.77 D), and HO_2_^•^ (with a dipole moment of 2.23 D) might be permeable. The candidates that can escape from the mitochondria include ROS with small dipole moments, i.e., H_2_O_2_, NO^•^, NO_2_^•^, HO_2_^•^, ONOOH, ^•^OH, and H_2_O. It is well-known that NO_2_^•^ reacts with urate, ascorbate, and GSH at 10^7^ M^–1^ S^–1^ [55]. Therefore, the reaction of NO_2_^•^ with specific targets in the cytoplasm, where GSH is present at µM–mM levels [56,57], likely occurs with very low frequency [58,59]. Short-lived molecules, such as ^•^OH, are unlikely to contribute to intracellular signaling.

Based on considerations of membrane permeability, chemical stability, and intracellular reactivity, H_2_O_2_, HO_2_^•^, ONOOH, and NO^•^ emerge as the most plausible candidates for mediating mitochondria-to-cytosol redox communication [49]. These species may act as diffusible messengers that transmit mitochondrial redox status to other cellular compartments and thereby contribute to the activation of intracellular signaling pathways.

### 5.2. Evidence for Mitochondria-Initiated Signal Transduction

To investigate the role of mtROS in signal transduction, we modulated mitochondrial superoxide levels through the overexpression of human MnSOD (hMnSOD). Reduction in mitochondrial superoxide levels resulted in decreased expression of multiple components of the signaling pathway, including nuclear factor erythroid 2-related factor 2 (Nrf2), Kelch-like ECH-associated protein 1 (Keap1), heme oxygenase-1 (HO-1), heme oxygenase-2 (HO-2), MnSOD, glutamate–cysteine ligase (GCL), glutathione S-transferase (GST), and nicotinamide adenine dinucleotide phosphate (NAD(P)H) quinone oxidoreductase 1 (NQO1) [60]. These findings suggest that mtROS contribute to the activation of the Nrf2/Keap1 pathway and can regulate transcriptional responses in the nucleus.

Further evidence for mitochondria-initiated signaling was obtained from studies of GATA transcription factors. We demonstrated that reduction in mitochondrial superoxide levels by MnSOD overexpression significantly decreased the expression of GATA1, GATA3, GATA4, and GATA5 [61]. Because GATA3 is known to be regulated by nuclear factor-kappa B (NF-κB) signaling [62], these findings suggest that mtROS also influence NF-κB-dependent signaling pathways.

In cells, signal transduction networks act to maintain homeostasis and prevent major changes in intracellular status, including alterations to redox potentials. Among the multiple pathways involved, signal transduction via NF-κB appears to play a key role during inflammation, immunity, development, cell growth, and survival. NF-κB regulates over 100 genes, including those with both antioxidant and pro-oxidant functions [60,61]. Tumor necrosis factor-α (TNF-α) is a well-established inducer of NF-κB, and induction occurs in a ROS-dependent manner [63]. Although antioxidant TNF-α has been reported to induce NF-κB activation [64], there exists overwhelming evidence for a key role of numerous oxidants in this process, which is postulated to be clinically important in the manifestation of several diseases [65,66,67,68,69,70,71,72,73,74]. The protein NF-κB essential modulator (NEMO, known as an inhibitor of NF-κB kinase subunit gamma, IKK-γ) is a subunit of the IκB kinase complex that activates NF-κB when present in a dimeric (disulfide-bonded) form. The formation of these disulfide bonds involves Cys54 and Cys347, and the treatment of cells with hydrogen peroxide enhances the formation of NEMO dimers. These findings suggest that oxidants can activate the NF-κB-related systems [75].

**Figure 4:**
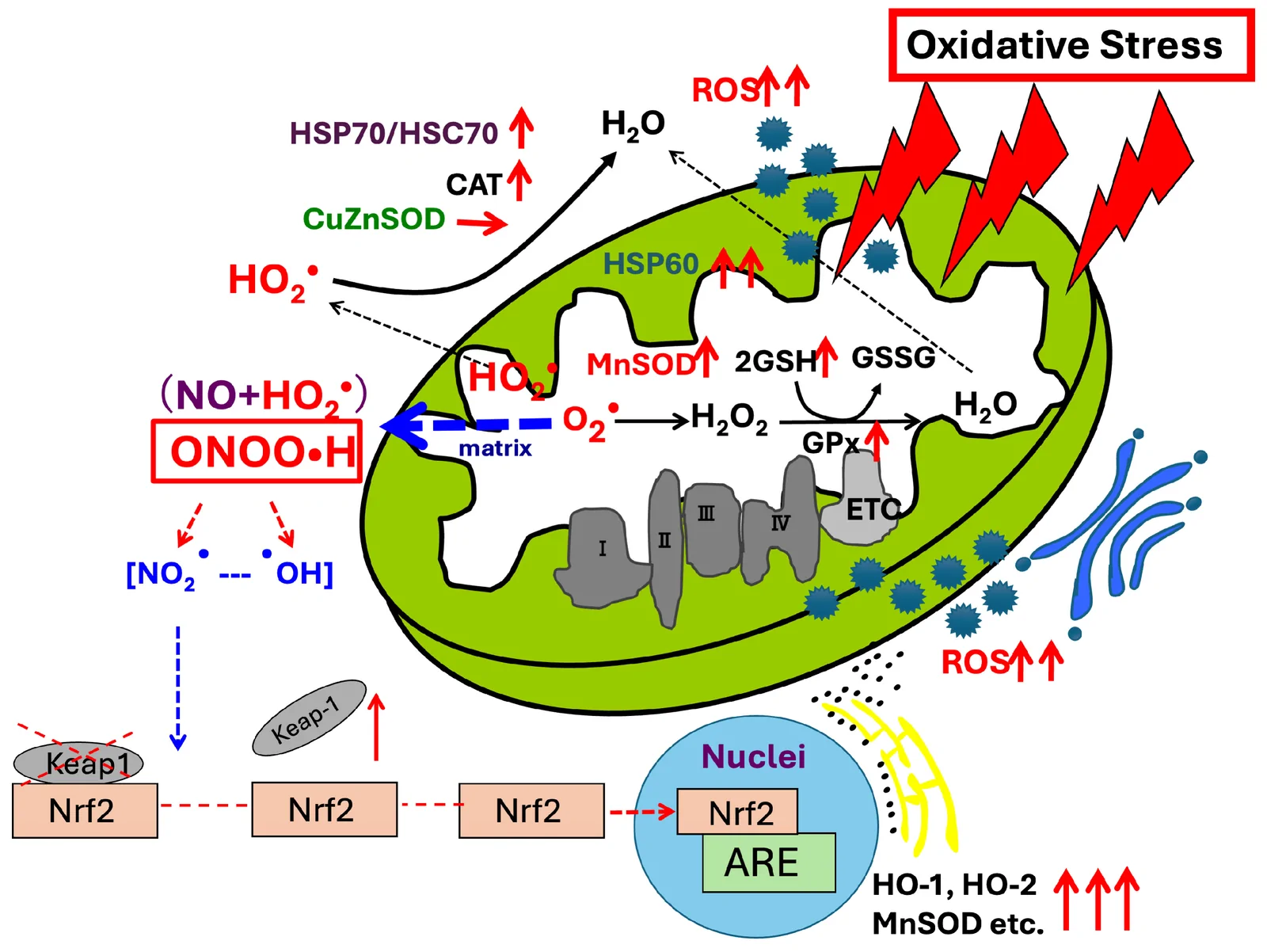
**A model of mitochondrial ROS-mediated activation of intracellular signaling pathways.** Oxidative stress increases electron leakage from the mitochondrial electron transport chain (ETC), resulting in the generation of superoxide (O_2_^•−^) and downstream ROS. Following conversion by mitochondrial antioxidant enzymes, including MnSOD and glutathione peroxidase (GPx), diffusible ROS such as hydrogen peroxide (H_2_O_2_), hydroperoxyl radical (HO_2_^•^), and peroxynitrous acid (ONOOH) may exit mitochondria and participate in intracellular signaling. Oxidative modification of Keap1 promotes the release and nuclear translocation of Nrf2, leading to activation of antioxidant response element (ARE)-dependent gene expression, including HO-1, HO-2, MnSOD, and other antioxidant proteins. This model illustrates how mitochondrial ROS function as signaling mediators linking oxidative stress to nuclear gene regulation and cellular adaptation responses. Adapted from Indo et al. 2023 [60].

By modulating redox-sensitive pathways, including the Nrf2/Keap1 [60] and NF-κB [61] signaling pathways, mtROS occupy a central position in the regulation of cellular homeostasis, stress responses, and gene expression. These observations provide experimental support that mitochondria serve as signaling organelles capable of initiating intracellular signal transduction through ROS-dependent mechanisms (Figure 4).

## 6. Finding of HSP47 Localization in Mitochondria

### 6.1. Subcellular Localization of HSP47

HSP47, also known as serpin family H member 1 (SERPINH1), has long been recognized as an ER-resident molecular chaperone that plays an essential role in collagen biosynthesis and protein folding within the ER lumen [76,77]. Under physiological conditions, HSP47 is predominantly localized in the ER and is widely regarded as an ER-specific stress-response protein. HSP47 binds with collagen with the biding motifs. In Table 1 the predicted HSP47-binding motifs are shown. These motifs can be used for proteins even outside ER to bind HSP47.

### 6.2. Human HSP47 Overexpression Causes ROS Generation in Cells

**Figure 5:**
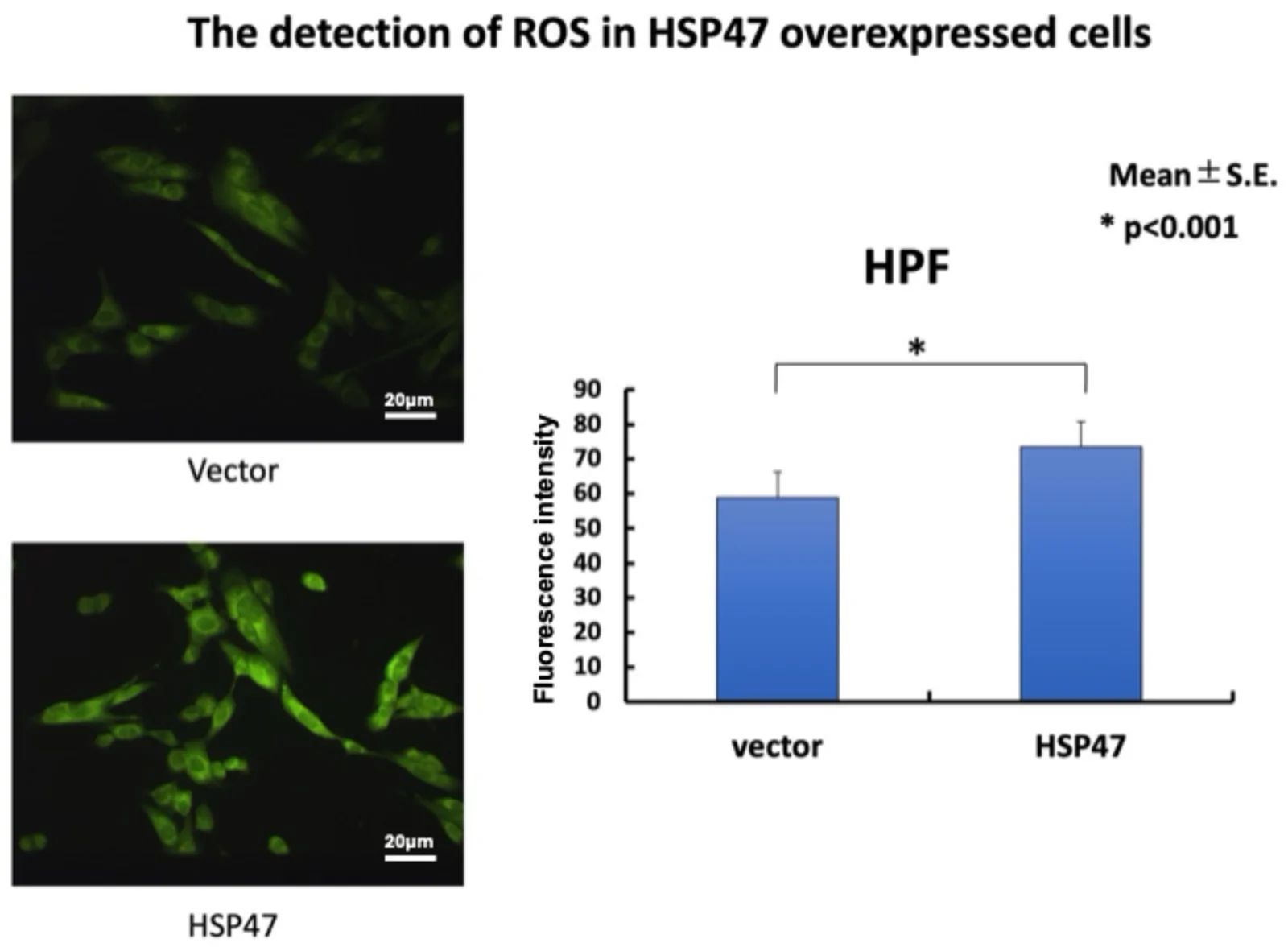
**HSP47 overexpression enhances intracellular ROS production.** Human neuroblastoma SK-N-SH cells were transfected with either an empty vector or an HSP47 expression construct. Intracellular ROS levels were detected using the ROS-sensitive fluorescent probe hydroxyphenyl fluorescein (HPF). Representative fluorescence images are shown on the left. Quantitative analysis demonstrated significantly increased ROS production in HSP47-overexpressing cells compared with vector controls (*p* < 0.001). Modified from Indo et al. 2021 [85].

mtROS play central roles in oxidative stress, apoptosis, and intracellular signal transduction. To investigate the relationship between HSP47 and mtROS production, Indo et al. examined the effects of irradiation-induced cellular stress in human neuroblastoma SK-N-SH cells [85]. Exposure to electron-beam or X-ray irradiation (10–15 Gy) resulted in a significant increase in mtROS generation, accompanied by enhanced lipid peroxidation and translocation of HSP47 from the ER to mitochondria [85]. Furthermore, overexpression of HSP47 resulted in increased mtROS [85]. In Figure 5, the detection of ROS in HSP47-overexpressing cells is shown. Previously, Indo et al. proved that HPF fluorescence measured around mitochondria originates from mitochondria using MnSOD gene transfection. MnSOD is located in mitochondria and scavenges superoxide [38]. Therefore, the fluorescence shown in Figure 5 is localized in mitochondria. These findings suggest a close association between mitochondrial localization of HSP47 and increased oxidative stress within the organelle (Figure 5). These results indicate that HSP47 is not merely a marker of cellular stress but may actively participate in the regulation of mitochondrial redox homeostasis.

In addition to increased ROS production, irradiation-induced HSP47 translocation was associated with elevated lipid peroxidation, suggesting that HSP47-mediated oxidative stress may influence mitochondrial membrane integrity and function [85]. Because mtROS are known to regulate both apoptosis and intracellular signaling pathways, the ability of HSP47 to enhance mtROS generation provides a potential mechanistic link between ER stress, mitochondrial dysfunction, and downstream cellular responses.

The molecular mechanism by which HSP47 promotes mtROS production remains to be fully elucidated. However, the stress-induced translocation of HSP47 to mitochondria, together with the observed increase in ROS generation following HSP47 overexpression, strongly suggests that HSP47 participates in the regulation of mitochondrial redox processes [85]. These findings challenge the traditional view of HSP47 as an exclusively ER-resident chaperone and support a broader role for HSP47 in mitochondrial biology.

The available evidence suggests that HSP47 translocation to mitochondria represents a previously unrecognized mechanism for regulating mtROS generation. Through its effects on mitochondrial oxidative stress, HSP47 may influence apoptosis, redox signaling, and cellular adaptation to stress, thereby linking ER–mitochondrial communication to multiple physiological and pathological processes.

## 7. Virus–Mitochondrial Interactions and Hypothetical Roles of HSP47

### 7.1. Interactions Between Viruses and Mitochondria

Mitochondria are increasingly recognized as critical targets of viral infection. Recent work by Gong et al. showed that Newcastle disease virus degrades SIRT3 via PINK1-PRKN-dependent mitophagy [86]. Several proteins from human CMV (HCV) have been shown to traffic from the ER to mitochondria through mitochondria-associated membranes (MAMs), specialized contact sites that facilitate communication between the ER and mitochondria [87]. Ohta & Nishiyama (2011) demonstrated that viral proteins localize to mitochondria and interact with mitochondrial proteins to regulate host-cell responses [88]. Members of the genus Mitovirus, family *Narnaviridae*, are composed of a single genome segment of positive-sense RNA that encodes only RNA-dependent RNA polymerase (RdRp) [89,90]. Mitoviruses localize to the mitochondria and, in the case of plant pathogenic fungi, can attenuate the virulence of the fungi by impairing mitochondrial function [91]. Namgaladze et al. (2019) reported ER–mitochondria communication linked to inflammatory and ER stress responses and its roles in apoptotic cell engulfment, activation of the inflammasome, and antiviral defense [92]. Elesela and Lukacs (2021) reported that viruses affect mitochondrial functions and impact mitochondrial metabolism and innate immune signaling [93]. Sorouri et al. (2022) noted that new dimensions of mitochondrial biology are emerging as battlefronts during viral infection, while apoptosis and type I interferon signaling (IFN-I) mediated by mitochondrial antiviral signaling (MAVS) are well-established defenses [94]. Many viruses manipulate mitochondrial respiratory and apoptotic functions, and viral interactions with mitochondria lead to increased production of ROS [95]. Several viral proteins specifically target mitochondria to subvert host defense. By targeting mitochondria, these viruses have acquired the capability to control apoptosis and metabolic state and evade immune responses in host cells [96].

Mitochondria can be an attractive therapeutic target by limiting energy for viral replication [97]. Viral pathogens have evolved sophisticated mechanisms to manipulate mitochondrial morphology and function to facilitate their replication [98]. Viruses have developed sophisticated mechanisms to target and disrupt mitochondrial function, which contribute to cognitive decline and neurodegeneration [99]. Many viral proteins target mitochondria, controlling mitochondrial morphology, metabolism, and immune response, thereby achieving immune evasion, promoting their proliferation, and accelerating the infection process [100].

Recent studies have focused on the possible roles of mitochondria in SARS-CoV-2 infection [101]. We recently hypothesized that lipidated (prenylated/myristoylated) SARS-CoV-2 proteins target host mitochondria, inducing mtROS and mitochondrial DNA (mtDNA) damage to drive long COVID pathology [101]. It has been suggested that mitochondrial hijacking by SARS-CoV-2 could be a key factor in COVID-19 pathogenesis [102]. SARS-CoV-2 aberrantly elevates mitochondrial bioenergetics and activates the EGFR-mediated cell survival signal cascade during the early stage of viral infection. SARS-CoV-2 causes an increase in mitochondrial transmembrane potential via the SARS-CoV-2 RNA–nucleocapsid cluster [103]. Many mitochondrial dysfunctions persist after recovery from viral infections, and quantitative defects and structural rearrangements of mitochondrial DNA accumulate in post-mitotic tissues long after SARS-CoV-2 or HIV infection [104].

### 7.2. Virus Localization to Mitochondria, mtROS Generation, and mtDNA Damage; Hypothetical Roles of HSP47

Mitochondria regulate numerous cellular processes, including ATP production, apoptosis, innate immune signaling, and redox homeostasis [9,10,101,105]. Because mitochondria are a major source of cellular reactive oxygen species [38], viral interactions with mitochondria can profoundly influence mtROS production and downstream cellular responses.

HSP47 has recently been shown to translocate from the ER to mitochondria under oxidative stress, resulting in enhanced mtROS generation [85]. HSP47 may represent a previously unrecognized mediator linking virus-induced ER stress to mitochondrial dysfunction. RNA viruses, particularly positive-sense RNA viruses such as SARS-CoV-2 and HCV, induce ER stress by remodeling ER membranes to generate replication organelles [106] and by producing large amounts of viral glycoproteins. These alterations activate the unfolded protein response (UPR) and alter the expression and intracellular localization of ER-resident chaperones, including GRP78/BiP (heat shock protein family A (Hsp70) member 5) [107,108]. In contrast, DNA viruses are more directly associated with activation of cytosolic DNA-sensing pathways, particularly the cGAS–STING axis [109,110]. HSP47 may indirectly modulate antiviral innate immunity by linking virus-induced ER stress to mitochondrial oxidative stress and mtDNA release. Cytosolic mtDNA subsequently activates the cGAS–STING pathway, leading to TBK1–IRF3 activation, type I interferon production, and inflammatory responses [109,110]. Direct experimental evidence supporting this hypothesis is currently lacking, and further studies are required to validate the involvement of HSP47 in virus-induced ER–mitochondria communication and antiviral innate immune responses.

## 8. Conclusions and Future Perspectives

**Figure 6:**
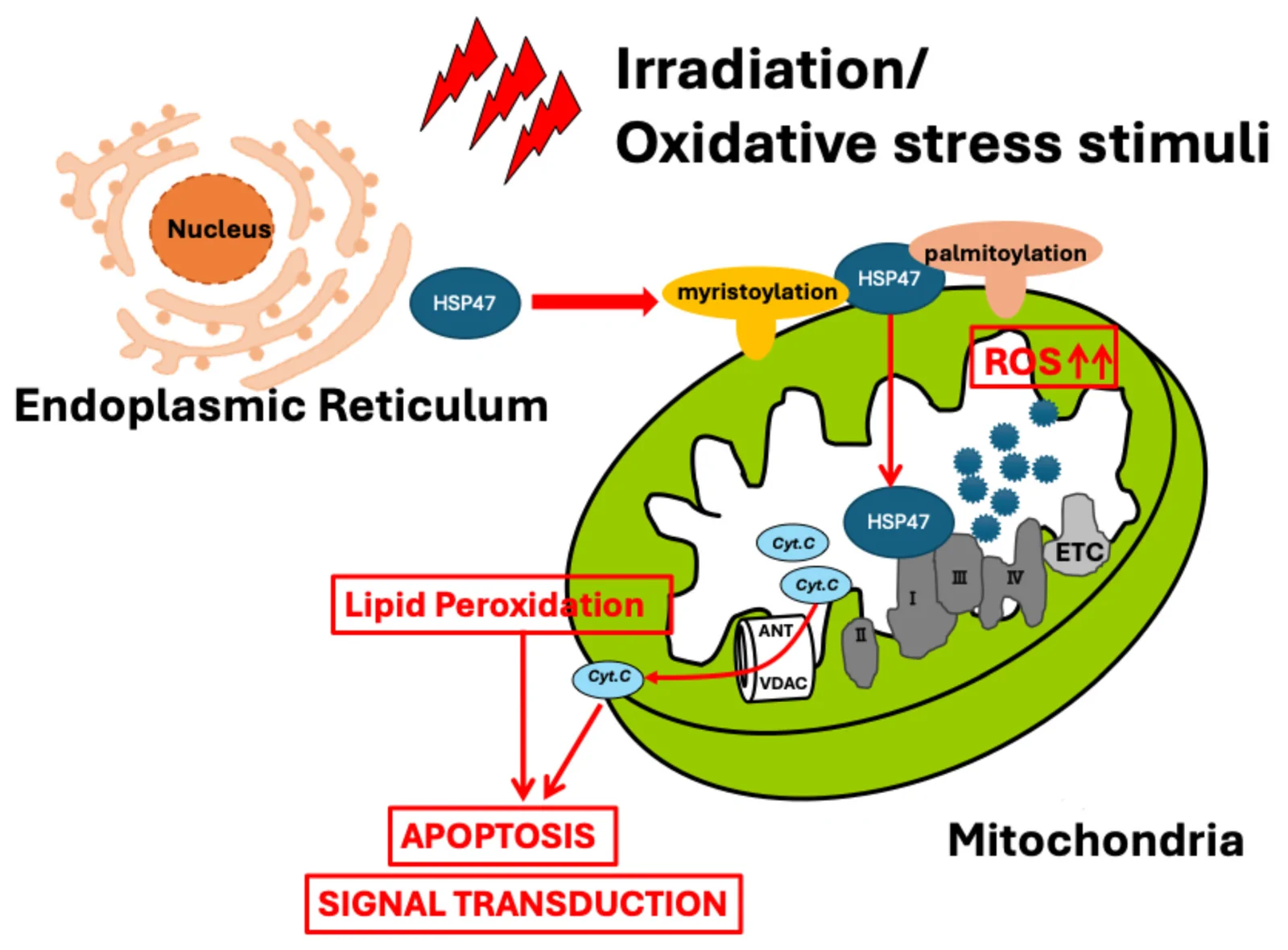
**HSP47-mediated mtROS generation and downstream cellular responses.** Under conditions of oxidative stress, including irradiation-induced stress, HSP47 translocates from the endoplasmic reticulum (ER) to mitochondria. Bioinformatic analyses suggest that lipid-mediated membrane interactions, including putative myristoylation- and palmitoylation-associated mechanisms, may contribute to this intracellular trafficking process. Following mitochondrial localization, HSP47 is associated with increased mtROS generation. Elevated mtROS promote lipid peroxidation, mitochondrial dysfunction, and cytochrome c release, leading to apoptosis. In parallel, mtROS may activate intracellular signaling pathways that regulate cellular stress responses.

Mitochondrial reactive oxygen species (mtROS), while inherent byproducts of electron transport chain activity, serve as crucial regulators of both apoptosis and cellular signaling pathways. However, the precise mechanisms governing these processes remain to be fully elucidated. Recent findings by Indo et al. (2021) highlighted a novel regulatory axis wherein the endoplasmic reticulum-resident protein, HSP47, translocates to the mitochondria and promotes mtROS generation under specific oxidative stress conditions [85]. Deciphering the exact molecular mechanisms behind HSP47 translocation and its subsequent regulation of mtROS will advance our fundamental understanding of mitochondrial biology. Ultimately, targeting the HSP47–mtROS axis may offer novel therapeutic avenues for oxidative stress-related diseases. Furthermore, although direct evidence is currently limited, investigating whether this mechanism extends to virus-induced ER–mitochondrial communication represents an important direction for future research (Figure 6).

## Figures and Tables

**Table 1:** Predicted HSP47-binding motifs.

HSP47 Binding Motif Residues	References
RDEL: Arg-Asp-Glu-Leu	Clarke E.P. and Sanwal, B.D., 1992 [78]
PPG(n): Pro-Pro-Gly(n)	Koide T et al., 1999 [79]
XRG: Xaa-Arg-Gly	Koide T et al., 2002 [80]
YXXR:Tyr-Xaa-Xaa-Arg	Koide T et al., 2006 [81]
X(T/P)GXRG: Xaa-(Thr/Pro)-Gly-Xaa-Arg-Gly	Nishikawa Y et al., 2010 [82]
XRG: Xaa-Arg-Gly, XDG: Xaa-Asp-Gly	Widmer C et al., 2012 [83]
GXR: Gly-Xaa-Arg	Cai H et al., 2021 [84]

**Predicted HSP47-interacting motifs.** Putative HSP47-binding motifs identified by sequence analysis are highlighted.

## Data Availability

No new data were created or analyzed in this study. Data sharing is not applicable to this article.

## Abbreviations

The following abbreviations are used in this manuscript:

Bcl-2	B-cell/CLL lymphoma 2
BiP	Binding immunoglobulin protein
cGAS	Cyclic GMP-AMP synthase
CMV	Cytomegalovirus
CMXRos	Chloromethyl-X-rosamine
COVID-19	Coronavirus disease 2019
ΔΨ (Delta Psi)	Mitochondrial membrane potential
DNA	Deoxyribonucleic acid
EGFR	Epidermal growth factor receptor
ER	Endoplasmic reticulum
ETC	Electron transport chain
GCL	Glutamate–cysteine ligase
GPx	Glutathione peroxidase
GPx4	Phospholipid hydroperoxide glutathione peroxidase
GRP78	78 kDa glucose-regulated protein
GST	Glutathione S-transferase
HCV	Human CMV
HIV	Human immunodeficiency virus
HNE	4-Hydroxynonenal
H_2_O_2_	Hydrogen peroxide
HO	Heme oxygenase
HO_2_^•^	Hydroperoxyl radical
HPF	hydroxyphenyl fluorescein
HSP	Heat shock protein
IRF3	Interferon regulatory factor 3
Keap1	Kelch-like ECH-associated protein 1
MAMs	Mitochondria-associated membranes
MAVS	Mitochondrial antiviral-signaling protein
MIA	Mitochondrial intermembrane space assembly
MnSOD	Manganese superoxide dismutase
MPP	Mitochondrial processing peptidase
mtDNA	Mitochondrial DNA
mtROS	Mitochondrial reactive oxygen species
MTS	Mitochondrial targeting sequence
NADPH	Nicotinamide adenine dinucleotide phosphate
NEMO	NF-κB essential modulator
NF-κB	Nuclear factor-kappa B
NO^•^	Nitric oxide
NO_2_^•^	Nitrogen dioxide
NQO1	NAD(P)H quinone oxidoreductase 1
Nrf2	Nuclear factor erythroid 2-related factor 2
O_2_^•−^	Superoxide anion
^•^OH	Hydroxyl radicals
^1^O_2_	Singlet oxygen
ONOO^−^	Peroxynitrite
ONOOH	Peroxynitrous acid
PAM	Presequence translocase-associated motor
PINK1	PTEN-induced kinase 1
PRKN	Parkin RBR E3 ubiquitin protein ligase
RdRp	RNA-dependent RNA polymerase
RNA	Ribonucleic acid
RNS	Reactive nitrogen species
ROS	Reactive oxygen species
rRNAs	Ribosomal RNAs
SAM	Sorting and assembly machinery
SARS-CoV-2	Severe acute respiratory syndrome coronavirus 2
SIRT	Sirtuin
SOD	Superoxide dismutase
SRP	Signal recognition particle
STING	Stimulator of interferon genes
TANK	TRAF family member-associated NF-kappa-B activator
TBK1	TANK-binding kinase 1
TIM	Translocase of the inner membrane
TOM TRAF	Translocase of the outer membrane Tumor necrosis factor receptor-associated factor
tRNAs	Transfer RNAs
UPR	Unfolded protein response
